# Structural alterations in the prefrontal cortex mediate the relationship between Internet gaming disorder and depressed mood

**DOI:** 10.1038/s41598-017-01275-5

**Published:** 2017-04-28

**Authors:** Jihye Choi, Hyun Cho, Jin-Young Kim, Dong Jin Jung, Kook Jin Ahn, Hang-Bong Kang, Jung-Seok Choi, Ji-Won Chun, Dai-Jin Kim

**Affiliations:** 1 Department of Psychiatry, Seoul St. Mary’s Hospital, The Catholic University of Korea College of Medicine, Seoul, Korea; 2 Department of Radiology, Seoul St. Mary’s Hospital, The Catholic University of Korea College of Medicine, Seoul, Korea; 3 Department of Digital Media, The Catholic University of Korea, Bucheon, Korea; 4 Department of Psychiatry, SMG-SNU Boramae Medical Center, Seoul, Korea

## Abstract

Adaptive gaming use has positive effects, whereas depression has been reported to be prevalent in Internet gaming disorder (IGD). However, the neural correlates underlying the association between depression and Internet gaming remain unclear. Moreover, the neuroanatomical profile of the striatum in IGD is relatively less clear despite its important role in addiction. We found lower gray matter (GM) density in the left dorsolateral prefrontal cortex (DLPFC) in the IGD group than in the Internet gaming control (IGC) group and non-gaming control (NGC) group, and the GM density was associated with lifetime usage of Internet gaming, depressed mood, craving, and impulsivity in the gaming users. Striatal volumetric analysis detected a significant reduction in the right nucleus accumbens (NAcc) in the IGD group and its association with lifetime usage of gaming and depression. These findings suggest that alterations in the brain structures involved in the reward system are associated with IGD-related behavioral characteristics. Furthermore, the DLPFC, involved in cognitive control, was observed to serve as a mediator in the association between prolonged gaming and depressed mood. This finding may provide insight into an intervention strategy for treating IGD with comorbid depression.

## Introduction

Playing Internet games has recently become a popular activity^1^. While the adaptive use of Internet gaming improves spatial cognition^2–5^ and functions as entertainment, prolonged exposure to and a loss of control over Internet gaming have repercussions for an individual’s emotion, cognition and behavior^6–11^. Compulsive and uncontrolled use of Internet gaming has become an emerging issue in mental health all over the world; therefore, Internet gaming disorder (IGD) was recently introduced in Section 3 of the fifth edition of the Diagnostic and Statistical Manual of Mental Disorders (DSM-5) in 2013^12^.

Previous neuroimaging studies have suggested that IGD shares similar neurobiological mechanisms to addiction such as abnormal fronto-striatal networks, which are involved in reward processing and cognitive control^13–16^. Structurally, gray matter (GM) volume and cortical thickness in prefrontal areas including the dorsolateral prefrontal cortex (DLPFC) and striatum were correlated with addiction duration, gaming duration, cognitive deficits and severity of IGD^17–19^. Functionally, the abnormal involvement of the fronto-striatal network has been associated with impairments in inhibition^20–22^, impulse control^23^, the severity of Internet addiction^24^, and affective and cognitive processing^25^. Moreover, altered activation in the frontal areas^26, 27^ and striatum^28^ in response to gaming cues have been reported in the IGD group. These findings are in accordance with the results of studies on pathological gambling and substance use disorder^29, 30^, supporting the emerging view that IGD is regarded as a type of behavioral addiction.

A large body of literature has reported a strong association of IGD, or Internet addiction, with depression in particular^6, 11, 24, 31–34^. These findings are mostly based on survey studies, and the neural correlates of this strong association have yet to be comprehensively identified. Even though individuals with a past or current psychiatric illness were excluded in the neuroimaging study, a higher level of depression was still observed in the IGD group in some studies^20, 28, 35–39^, which may potentially yield confounding effects. If comorbid depression reflects a psychological characteristic of IGD, the attempt to explore the neurobiological substrates associated with the relationship between IGD and depressed mood would broaden the therapeutic approach with the improved understanding of IGD, as also mentioned by Tam^40^.

The striatum plays a significant role in reward and motivational processing, and its abnormalities are implicated in neuropsychiatric diseases such as addiction and depression^41, 42^. Despite its essential role in addiction, the neuroanatomical characteristics of the striatum are relatively less investigated in IGD research except for two studies conducted by a team of researchers^19, 20^. These studies reported increased volume of the caudate nucleus and nucleus accumbens (NAcc), which were associated with cognitive control and the severity of the addiction, respectively. Because the subjects of these studies were adolescents and young adults and included females, we attempted to study males in their 20 s and 30 s including non-gaming users in the current study.

We conducted the current study on a sample consisting of Internet gaming users, divided into the IGD and IGC (Internet gaming control) groups, and non-gaming users. Past studies have only compared IGD and IGC (i.e., those who played but were not addicted to Internet gaming) groups. Therefore, adding the subjects who do not participate in Internet gaming, including mobile games, to the current study may provide a deeper insight into the gradual changes in the brain that occur along with the development of IGD. We used the voxel-based morphometry (VBM) method to detect neuroanatomical changes in an unbiased way across the whole brain and FreeSurfer software to measure the volume of the striatum. Furthermore, we explored whether the altered brain structures were associated with the IGD-related characteristics and whether the alterations influenced the relationship between prolonged Internet gaming and depression level in the Internet gaming users.

## Results

### Sample Characteristics

Table 1 summarizes the characteristics of the subjects. The three groups did not significantly differ in age and intelligence quotient (IQ). As subjects in the non-gaming control (NGC) group did not play Internet games, there were no other variables related to Internet gaming use. The IGD group exhibited higher IGD scores than the IGC group. The IGD group spent significantly more time playing Internet games weekly for the past one year than the IGC group, but lifetime usage of gaming was not different between the both groups, showing a trend toward significance (*P* = 0.055). Consistent with previous studies, the IGD group exhibited a higher level of depression than the IGC group even though our sample did not include any individuals with comorbidity. The craving for gaming and dysfunctional impulsivity were significantly higher in the IGD group than the IGC group.

**Table 1:** Sample characteristics.

Characteristics	IGD group (*n* = 22)	IGC group (*n* = 25)	NGC group (*n* = 24)	*P*-value
Age, years; mean (SD)	29.45 (4.74)	30.00 (5.75)	27.21 (4.88)	0.145
IQ; mean (SD)	111.45 (10.34)	117.76 (9.36)	112.17 (12.27)	0.089
IGD scale	6.27 (1.55)	0.60 (0.91)	NA	0.000*
SCL-90-R: Depression	21.59 (10.11)	9.6 (7.98)	NA	0.000*
Lifetime usage of IG, hours; mean (SD)	3916.76 (5052.46)	1473.55 (2274.82)	NA	0.055
Time for IG per week for the past one year, hours; mean (SD)	22.27 (9.78)	14.68 (7.72)	NA	0.005*
Craving for gaming; mean (SD)	5.57 (2.11)	3.90 (1.87)	NA	0.010*
DDII; mean (SD)	5.73 (2.33)	2.44 (3.07)	NA	0.000*

Abbreviations. DDII, Dickman’s Dysfunctional Impulsivity Inventory; IG, Internet gaming; IGC, Internet gaming control; IGD, Internet gaming disorder; IQ, intelligence quotient; NGC, non-gaming control; SCL-90-R, Symptom Checklist-90-Revised. *Significant at *P* < .05.

### MRI results

**Figure 1:**
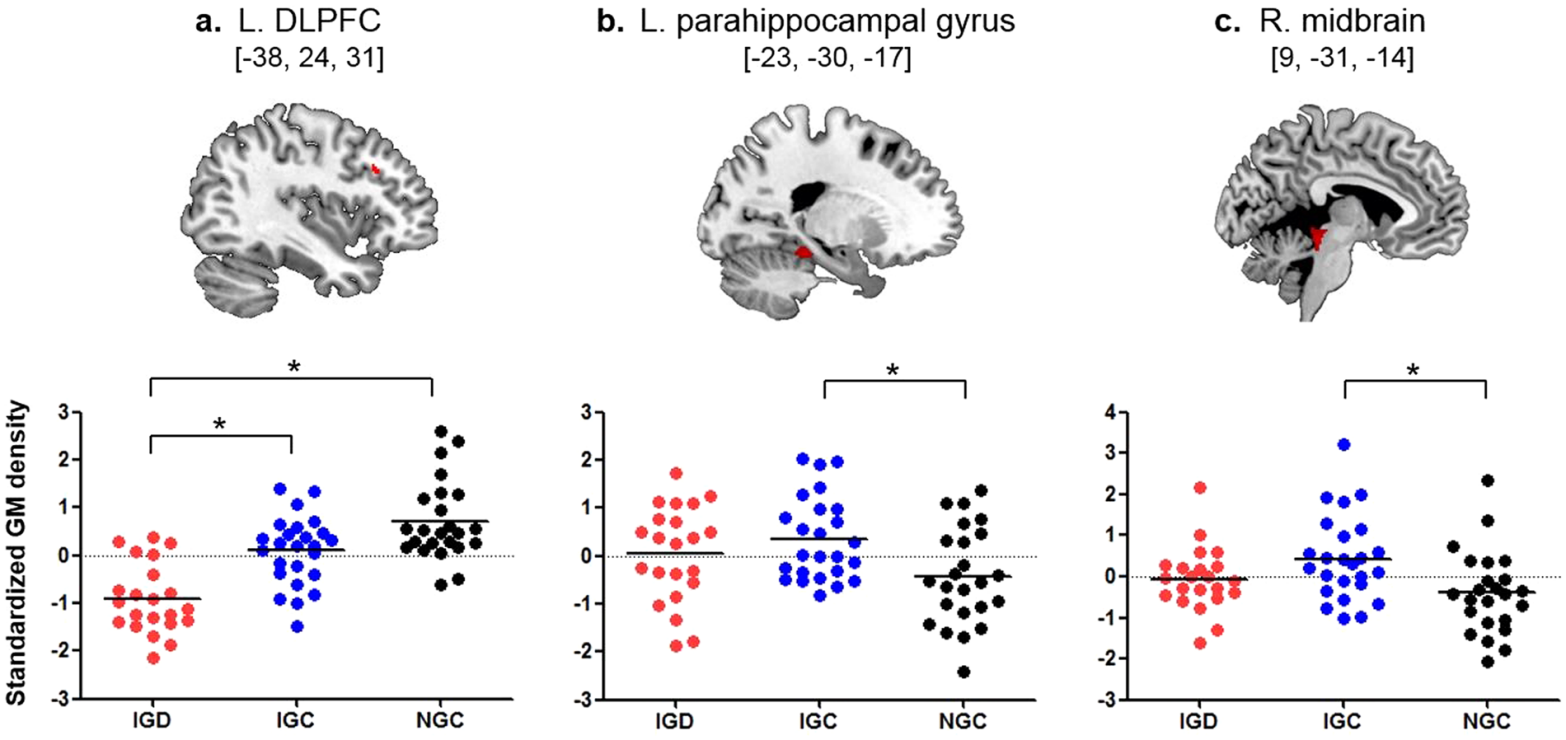
Voxel-wise comparisons among the Internet gaming disorder (IGD), Internet gaming control (IGC), and non-gaming control (NGC) groups. Standardized gray matter (GM) density was calculated for visualization. An analysis of covariance (ANCOVA) test detected the difference in (**a**) the left dorsolateral prefrontal cortex (DLPFC) among the three groups, and subsequent post hoc t-tests revealed the significant reduction in the IGD group compared with that in the IGC and NGC groups. The IGC group showed a higher GM density in a cluster covering (**b**) the parahippocampal gyrus and (**c**) midbrain than the NGC group. The results were adjusted for age and intelligence quotient (IQ). ^*^S﻿ignificant at *P* < 0.05.

Voxel-wise comparisons of the T1 images showed a GM density difference in the left dorsolateral prefrontal cortex (DLPFC) among the three groups [peak Montreal Neurological Institute (MNI) coordinates: −38, 24, 31; F_2, 66_ = 23.54]. Subsequent post hoc t-tests revealed that the IGD group showed a lower GM density in the left DLPFC than the IGC and NGC groups, while the IGC and NGC groups did not differ in the GM density in this region (Fig. 1). The IGC group showed a higher GM density in a cluster covering the parahippocampal gyrus and midbrain than the NGC group [peak MNI coordinates: −9, −33, −12; T_1, 66_ = 3.61] (Fig. 1). However, there was no region that showed a reduction in GM density in the IGC group compared with that of the NGC group. To ensure that the depression levels were not a confounding factor in the GM density difference between the Internet gaming user groups (e.g., IGD and IGC groups), we repeated the analyses by entering the score of the depression subscale of the Symptom Checklist-90-Revised (SCL-90-R) as a nuisance covariate. The finding of decreased GM density of the left DLPFC in the IGD group compared with that of the IGC group was still observed.

**Figure 2:**
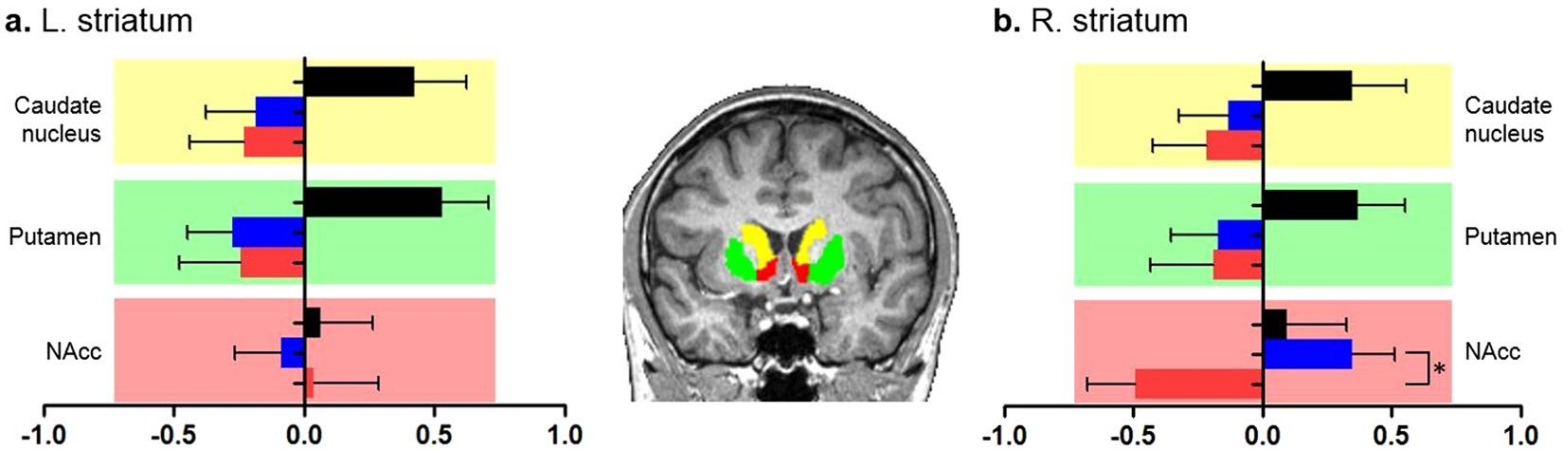
Comparisons of the standardized striatal volumes among the Internet gaming disorder (IGD), Internet gaming control (IGC), and non-gaming control (NGC) groups. (**a**,**b**) Although the volumes of the bilateral caudate nucleus and putamen were not significantly different among three groups, (**b**) the volume in the right nucleus accumbens (NAcc) was different, which was driven by the smaller volume in the IGD group than that in the IGC group, adjusting for age and the estimated total intracranial volume (eTIV). In the brain image, each color represents a brain region (yellow: the caudate nucleus, green: putamen, red: nucleus accumbens). The colors of the bar graphs indicate the following: black, NGC group; blue, IGC group; red, IGD group. The outputs of the striatal areas, which were obtained from FreeSurfer, are overlaid on the brain image of a subject. ^*^S﻿ignificant at *P* < 0.05.

Table 2 and Figure 2 represent the striatal volumes obtained from FreeSurfer. The estimated total intracranial volume (eTIV) was different among the three groups (*P* = 0.013) but not between the Internet gaming user groups (*P* = 0.430). Although the volumes of the bilateral caudate nucleus and putamen were not significantly different among three groups (Table 2; left caudate nucleus, *P* = 0.795; left putamen, *P* = 0.126; right caudate nucleus, *P* = 0.987; right putamen, *P* = 0.833), Figure 2 illustrates a distinct difference between the Internet gaming groups and NGC group by presenting the results of the comparisons of the standardized striatal volumes. The Internet gaming groups showed negative values in the volumes of the bilateral dorsal striatum, consisting of the caudate nucleus and putamen, compared with the NGC group that showed positive values. We found that the volume of the right NAcc, adjusting for age and eTIV, was significantly different among the three groups, and this difference still survived a more stringent correction for multiple comparisons. A post hoc analysis revealed that this volumetric difference was driven by the smaller volume in the IGD group than that in the IGC group.

**Table 2:** The volumes of the striatum.

	IGD group (n = 22)	IGC group (n = 25)	NGC group (n = 23)	*P*-value	Post hoc
*Left*					
Caudate nucleus	3659.62 (471.73)	3679.81 (449.48)	3969.88 (462.14)	0.795	
Putamen	5992.81 (816.89)	5971.80 (643.29)	6567.08 (627.80)	0.126	
NAcc	621.82 (112.02)	609.77 (84.98)	624.17 (93.06)	0.617	
*Right*					
Caudate nucleus	3586.33 (474.86)	3628.10 (466.38)	3859.10 (482.89)	0.987	
Putamen	5757.15 (727.03)	5767.84 (578.56)	6114.25 (555.94)	0.833	
NAcc	591.23 (90.63)	676.14 (83.71)	650.40 (113.80)	0.008*	IGC > IGD
eTIV	1511.88 (156.36)	1544.87 (136.81)	1635.62 (133.74)	0.013*	NGC > IGD, IGC

Abbreviations: eTIV, estimated total intracranial volume; IGC, internet gaming control; IGD, internet gaming disorder; NAcc, nucleus accumbens; NGC, non-gaming control. ^*^S﻿ignificant at *P* < 0.05.

### Relationship between the structural measurement and IGD characteristics

The correlation analyses were performed merging the IGD and IGC groups together to explore the relationship between the structural measurements and IGD-related characteristics (Table 3). The GM density in the left DLPFC, detected in the VBM analysis, was negatively correlated with IGD severity, lifetime usage of Internet gaming, depression, craving, and impulsivity but not with weekly Internet gaming time. However, the volume of the right NAcc, obtained from the FreeSurfer segmentation, was negatively associated with lifetime usage of Internet gaming and depressed mood, but the association with IGD severity, weekly Internet gaming time, craving, and impulsivity did not reach statistical significance.

**Table 3:** The relationship between the volumetric measurements and IGD characteristics.

	GM density in the left DLPFC		Volume in the right NAcc^a^	
	*r*	*P*-value	*r*	*P*-value
IGD severity	−0.415	0.004*	−0.312	0.053
Depression	−0.463	0.001*	−0.394	0.013*
Time for IG per week for the past year	−0.032	0.829	−0.128	0.438
Lifetime usage of IG	−0.392	0.011*	−0.402	0.011*
Craving	−0.419	0.006*	−0.151	0.339
Impulsivity	−0.354	0.015*	−0.050	0.731

Abbreviations: DLPFC, dorsolateral prefrontal cortex; GM, gray matter; IG, Internet gaming; IGD, Internet gaming disorder; NAcc, nucleus accumbens. ^a^A partial correlation analysis was conducted, adjusting for age and estimated total intracranial volume (eTIV) as covariates. ^*^Significant at *P* < 0.05.

**Figure 3:**
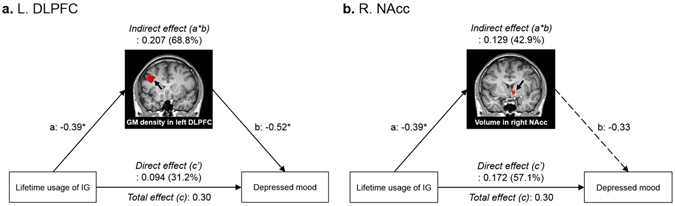
Mediation analysis to explore the neural substrates influencing the relationship between the lifetime usage of Internet gaming and depression. Structural alterations in (**a**) the left dorsolateral prefrontal cortex (DLPFC), not (**b**) right nucleus accumbens (NAcc), showed a mediating effect on the association between prolonged Internet gaming and depressed mood in the Internet gaming users. ^*^Significant at *P* < 0.05.

Furthermore, we conducted a mediation analysis to explore whether the structural alterations (mediator variable) influence the association between lifetime game usage (causal variable) and the self-reported depressed mood (outcome variable). Lifetime usage had an indirect influence on the level of depression through the alterations of the GM density in the left DLPFC, which bootstrap stimulation with 5,000 iterations confirmed to be statistically significant (indirect effect: 68.8%, 95% CI: 0.054, 0.389) (Fig. 3). However, the alteration of the volume in the right NAcc did not affect the relationship between lifetime usage of Internet gaming and the depressed mood in the Internet gaming users. These findings indicate that the left DLPFC, not the right NAcc, apprears to serve as a mediator in the association between prolonged Internet gaming use and depressed mood.

## Discussion

This is the first study, to our knowledge, to demonstrate the brain structure differences among IGD, IGC, and NGC groups. We also found that the observed brain alterations were associated with IGD characteristics. Furthermore, the current study showed the mediating effect of the altered brain structure on the relationship between lifetime usage of Internet gaming and the depressed mood in Internet gaming users.

Our findings demonstrate that the IGD group exhibited decreased GM density in the left DLPFC compared with the IGC and NGC groups. In the Internet gaming groups, lower GM density in the DLPFC was associated with more severe symptoms of IGD, more depressed mood, longer gaming time in life, more craving for gaming, and more impulsivity. These are consistent with the findings of previous neuroimaging studies, which stated that decreased GM density and dysfunction in the bilateral DLPFC in the IGD group compared with the IGC group were associated with the duration of addiction, gaming time, and craving for gaming^15–17, 43^. Additionally, we observed that the GM density in the left DLPFC did not differ between the IGC and NGC groups, who both were not addicted to the Internet gaming. The engagement of the DLPFC in IGD is not surprising given that the DLPFC plays a key role in the top-down control system that regulates behaviors and cognition (i.e., planning, motivation, decision making, and inhibitory control)^44^. Previous studies on the impaired reward system and addiction illustrate that the DLPFC is hyperactive in response to cue-induced craving and negative emotional stimuli, while it is hypoactive during cognitive tasks requiring inhibitory control^45, 46^. Structural anomalies in the DLPFC have also been frequently reported in patients that have difficulty controlling their behaviors and emotions, such as in substance abuse^47^, obsessive-compulsive disorder^48^ and depression^49^. On the basis of these findings, we can assume that the DLPFC abnormalities detected in the IGD group compared with the IGC and NGC groups may be responsible for the loss of behavioral control and the poor regulation of craving for gaming and negative emotion.

Although a large body of neuroimaging studies as well as survey studies have demonstrated the strong association between IGD and higher levels of depression, the attempts to link this association to changes in the brain related to IGD have yet to be comprehensively made. In the current study, the DLPFC was observed to serve as a mediator for the association between prolonged gaming in life and the self-reported depressed mood. In addition to the aforementioned role of the DLPFC in IGD, we suggest the possible involvement of the prefrontal dopaminergic system, which could be supported by several clinical studies on the efficacy of bupropion, an antidepressant, on IGD. For example, bupropion treatment mitigated gaming usage and craving for gaming with decreasing cue-induced activity in the left DLPFC in patients with IGD^27^, although the efficacy on depression is inconsistent in IGD with comorbid depression^50, 51^. Given that bupropion induces dopamine neurotransmission in both the prefrontal cortex and NAcc^52^ and its efficacy on other substance dependence with comorbid depression or attention-deficit hyperactivity disorder^53, 54^, it can be inferred that the prefrontal dopaminergic system may partly regulate negative mood as well as control impulsivity and craving for Internet gaming.

Dopamine plays a crucial role in the processing of salient information, such as gaming pictures^55^. Neurotransmitter signals between neurons such as dopamine influence the function and morphology of the neuronal circuit. Functionally, repeated exposure to salient stimuli affects dopaminergic pathways and decreases sensitivity to the natural stimuli, resulting in reward processing dysfunction^56, 57^. Repeated exposure also alters synaptic and structural morphology in the dendritic structures in brain areas involved in inhibitory control (i.e., the prefrontal cortex) and incentive motivation (i.e., the NAcc)^58, 59^. It is possible, therefore, that prolonged and persistent gaming in life may modulate dopaminergic functioning and produce morphological changes in the cell bodies or dendritic structures that lead to the DLPFC reduction, which is attributed to the poor regulation of depressed mood in the Internet gaming users.

This finding of the left DLPFC alteration may shed light on the therapeutic implications for IGD with comorbid depression. The issue about the lateralization of the DLPFC in IGD has not been investigated so far. Several functional studies reported the activation of the left or right DLPFC in response to gaming-cues^27, 60–62^, and Li *et al*.^63^ observed the positive relationship between the GM volume in the right DLPFC and Internet addiction score and cognitive inhibitory control in healthy young adults. These inconsistent results may be attributed to the different clinical variables examined in each study. However, the involvement of the left side DLPFC, which appears to be closely related to depressed mood in IGD in the current study, could be considered as a potential biomarker for IGD with comorbid depression based on clinical evidence from the repetitive transcranial magnetic stimulation (rTMS) over the DLPFC. The DLPFC stimulation modulates dopamine release in brain areas of the limbic system^64, 65^. Accumulating evidence suggests that the left DLPFC is more responsive to positive emotional information in healthy people, so that the stimulation over it in depressed people is known to enhance the response to positive stimuli by inducing cortical excitability in the left side^66–68^, whereas the right DLPFC is more responsive to negative emotional information and more involved in the cognitive modulation of emotional stimuli^66, 69^. In line with these findings, we could assume that individuals exposed to prolonged Internet gaming may be unable to respond to pleasant stimuli as appropriately as healthy individuals with the structural alteration in the left DLPFC, possibly yielding the high prevalence of comorbid depression in IGD. Thus, the left DLPFC can be considered as a potential biomarker for the depressed symptoms observed in IGD.

We observed smaller volumes of the bilateral dorsal striatum, consisting of the caudate nucleus and putamen, in the Internet gaming users compared with the NGC group. Although the absolute volumes were not significantly different, the relative comparison of the standardized values indicated a distinct volumetric change in the dorsal striatum in the Internet gaming groups compared with the NGC group. In drug addiction, the dorsal striatum receives efferent projections from the DLPFC that is associated with the inhibitory control and decision making, and thus impaired dopaminergic innervation to the dorsal striatum from the DLPFC is implicated in the failure of control over craving for salient stimuli^46, 70, 71^. This mechanism could provide grounds for the possible explanation for the change in the dorsal striatal volumes in the Internet gaming users compared with the non-gaming users. Prolonged Internet gaming that contains salient stimuli and rewarding effects may partly influence the capacity of self-regulation modulated by the DLPFC circuitry, which raises the risk of disruption of the dopaminergic projections to the dorsal striatum, gradually leading to increased craving for gaming and loss of control over gaming-seeking behavior that will develop the habitual and compulsive gaming use pattern. However, this assumption should be made cautiously because the addiction model is based on the drug addiction, and it is insufficient to account for the slight difference in the dorsal striatal volumes between the IGD and IGC groups. Hence, evidence on IGD needs to be more accumulated.

In contrast to the dorsal striatal volumes, the IGD group showed a statistically significant reduction in the right NAcc volume, when compared with the IGC group, but not with the NGC group. The drug-induced increase in dopamine release in the ventral striatum, where the NAcc is located, is associated with rewarding experiences such as pleasure but becomes blunted in the progress of addiction^71, 72^. Functional studies have revealed that addicted brains exhibit enhanced cue-induced activation in the nucleus accumbens and its association with craving^73, 74^. Similarly, IGD patients have also demonstrated increased right NAcc activation in response to gaming pictures and decreased functional connectivity to the midbrain, which was correlated with craving for gaming^36, 60^. There is another study that showed that increased cue-induced activation in the putamen was associated with smaller volume in the right NAcc^28^. These findings of hyperactivation of the NAcc in response to gaming-related cues and its association with craving for gaming suggest the important role of the NAcc in the control of motivation and reinforcement.

Nevertheless, the neuroanatomical profile of the striatum in IGD is relatively less clear despite the important role of the striatum in addiction, with the exception of two studies that showed increased volume of the right NAcc in IGD subjects compared with that of healthy controls^19, 20^. These conflicting results may be derived from the different sample characteristics. While their subjects were adolescents and young adults (aged 16–22 years) and included females, we studied males in their 20 s and 30 s.

We observed a significant reduction in the right NAcc in the IGD group, but the NAcc did not correlate with craving as in the aforementioned functional studies. Instead, the NAcc was negatively correlated with lifetime usage and depression scores. Interestingly, there were a couple of findings that showed that reduced volume of the NAcc was associated with a higher depression score in heroin users^75^ and lifetime cigarette smoking^76^. Volkow *et al*.^55^ suggested that striatal dopaminergic dysfunction may not be sufficient to account for addiction-related behaviors such as craving and impulsivity because other pathways involved in cognitive control and emotional regulation are likely to engage in the disrupted reward circuits that influence behavior characteristics. This suggestion could be corroborated by our finding that reduced DLPFC volume was associated with behavior traits that characterize addiction such as craving, impulsivity, and depression.

We also observed increased volumetric changes in the parahippocampal gyrus, midbrain and NAcc in the IGC group compared with that of the NGC group. One possible explanation for the increased changes in the midbrain and NAcc in the IGC group may be an inverted u-shaped relationship between dopamine levels and the cognitive performance and drug use^77^. For example, video gaming and cognitive training are associated with enhanced dopaminergic activity in prefrontal and striatal areas^78, 79^, and recreational cocaine users, not addicted to cocaine, had increased NAcc volume compared with that of the controls that was positively correlated with weekly usage^80^. The increased density of the parahippocampal gray matter may be potentially explained by the findings that healthy gaming is associated with changes in brain regions involved in spatial navigation, such as the parahippocampal gyrus^2–5^. Although it is not possible to link these structural changes to the cognitive capability or pleasurable experience due to a lack of related variables to test, it can be inferred that the GM growth in the gaming-related brain areas may reflect neuroadaptive plasticity indicative of positive effects of adaptive gaming use on the brain.

The current study has several limitations. First, our cross-sectional results should be interpreted with caution. We cannot determine whether the volumetric changes were induced by problematic Internet gaming because brain structural characteristics could be a precondition to indulge in Internet gaming. Thus, a longitudinal study may help to elucidate the development of IGD as well as the causal links among the volumetric changes, problematic Internet gaming, and behavioral characteristics. Second, the same depression scale was not administered to the NGC group. However, the purpose of the depression scale we used in the current study was different among the groups: we attempted to explore the neural basis influencing the relationship between Internet gaming use and depression in the IGD and IGC groups, and the NGC group, consisting of non-gaming users, had no variables related to Internet gaming use. With a different scale, we instead confirmed that no one in the NGC group was reported to be depressed.

In conclusion, the current study has shown that structural alterations in brain regions involved in cognitive control and reward processing are associated with IGD-related behavioral characteristics. Additionally, increased volumetric results in some brain regions observed in adaptive gaming users may provide an insight into the positive effects of adaptive Internet gaming use on the brain for future study. Notably, the left DLPFC appears to serve as a mediator in the association between prolonged Internet gaming use and depressed mood. This finding may broaden the therapeutic approach with the improved understanding of IGD.

## Methods

### Participants

Internet gaming users were recruited from 5,004 individuals who participated in an online survey on Internet gaming use. In the online survey, 2,935 people responded with interest in participating in the magnetic resonance imaging (MRI) study, and only males were selected because IGD is more prevalent in men than women. Of these people, males in their 20 s and 30 s who mostly played League of Legends (LOL), FIFA, or Sudden Attack were selected because these were the top three games played by those who responded to the survey. We divided Internet gaming users into two groups, Internet gaming disorder (IGD, *n* = 27) and Internet gaming control (IGC, *n* = 29) groups, on the basis of the clinician-administered interview and diagnostic criteria of IGD in the DSM-5 with cut-off scores of 5 or higher. Non-gaming users were recruited as the control (NGC, *n* = 26) group through advertisements on the college campus. Therefore, 82 males were recruited for the MRI study. We screened all individuals who reported current or past history of major medical, neurological, or psychiatric disorders, head injury, or metal implants that would preclude MRI scanning. All subjects were administered the Mini-International Neuropsychiatric Interview by a clinician to screen for psychiatric disorders: three subjects in the IGD group and two subjects in the IGC group were excluded from the analyses. Two subjects in the NGC group were excluded because their IQ was below 85, estimated by the short form of the Korean Wechsler Adult Intelligence Scale^81^. All subjects were high school graduates. They gave written informed consent approved by the Institutional Review Board of Seoul St. Mary’s Hospital in South Korea, by which all experimental protocols were approved. The methods were performed in accordance with the approved guidelines and regulations.

### Behavior Measures

#### Severity of IGD

The severity of IGD was assessed using the self-reported IGD scale investigating the 9 items described in the DSM-5: preoccupation, tolerance, withdrawal, persistence, escape, problems, deception, displacement, and conflict^12^. The IGD scale shows good criterion-related validity and reliability^82^.

#### Depressed mood

The level of depression in the Internet gaming users was assessed using the depression subscale of the SCL-90-R, although there was no participant with comorbidity. Previous studies have reported the association between depressed mood and IGD, as mentioned in the Introduction section. We, therefore, attempted to explore the neural substrates underlying this association. The SCL-90-R consists of 10 psychiatric symptom domains and includes a 13-item subscale for depression^83^. The reliability and validity of the Korean version of the SCL-90-R have been well established^84^. We confirmed that no one in the NGC group reported to be depressed by the Beck Depression Inventory^85^.

#### Internet gaming behaviors

We administered a questionnaire consisting of the following questions: “Which games do you play the most? ”; “How many hours have you participated in Internet gaming during weekdays and weekends on average for the last one year?”; “When did you begin playing Internet games, and how many hours have you played on a regular basis?”. On the basis of this information, the hours spent playing games per week for the last one year and lifetime usage of Internet gaming were computed. Moreover, the craving for gaming was obtained using a 10-point visual analog scale (1: not at all to 10: extreme).

#### Impulsivity

Impulsivity was assessed by the Dickman Dysfunctional Impulsivity Inventory (DDII)^86^. The Dickman Impulsivity Inventory (DII) evaluates dysfunctional and functional self-reported impulsivity, and we used the subscale of dysfunctional impulsivity, a tendency to act with less forethought, causing problems. The internal consistency coefficients for the two subscales in a sample of college students were 0.74 and 0.85, respectively. The ability to discriminate between functional and dysfunctional impulsivity was confirmed within the self-report domain of the Korean version of the DII^87^.

### MRI Acquisition

MRI data were acquired using a 3 Tesla Siemens MAGNETOM Verio scanner (Siemens, Erlangen, Germany) with an 8-channel sensitivity encoding (SENSE) head coil (SENSE factor = 2). Subjects’ heads were cushioned with attached earmuffs. The high-resolution T1-weighted magnetization-prepared rapid gradient echo (MPRAGE) images were collected with the following parameters: TR = 2,300 msec, TE = 2.22 msec, 176 slices, slice thickness = 1 mm, flip angle = 9°, voxel size = 1 × 1 × 1 mm, image matrix = 256 × 256, FOV = 256 mm^2^, and scan duration = 5 min 21 sec.

### Image Analysis

#### Voxel-based morphometry (VBM)

Preprocessing and VBM analysis were carried out using the VBM8 toolbox (http://dbm.neuro.uni-jena.de/vbm.html) in Statistical Parametric Mapping 8 (SPM8, Wellcome Department of Imaging Neuroscience, London, UK) implemented in Matlab R2011b (Mathworks, Sherborn, MA, USA). All image volumes were visually inspected by an investigator (JC) for artifacts and head motion. First, the origin of each subject’s T1 image was set on the anterior commissure (AC) and aligned along the anterior-posterior commissure line (AC-PC line). Images were segmented into tissue classes such as gray matter (GM), white matter (WM), and cerebrospinal fluid (CSF), which were affine registered to the tissue probability maps in the Montreal Neurological Institute (MNI) space. The affined-registered segments of all subjects were used to create the customized diffeomorphic anatomical registration through exponentiated lie algebra (DARTEL) template for the current study. Then, the GM tissue segment of each subject’s T1 image was spatially aligned to this template and then modulated for the non-linear components to locally preserve actual GM values by applying the correction for the brain size of the individual. The DARTEL-warped, normalized, non-linear-only modulated GM images were smoothed with a full-width half-maximum kernel of 8 mm. Before the statistical analysis, the resulting smoothed images were checked for homogeneity using covariance of the sample to detect the outlier. Two subjects in both the IGC and IGD groups were excluded from further analysis.

#### Volumetric segmentation of the striatum

Automated segmentation and labeling of the striatum was carried out using FreeSurfer software (version 5.1.0., http://surfer.nmr.mgh.harvard.edu), which utilizes a technique in which a neuroanatomical label is assigned to each voxel in an MRI image by estimating a probability distribution for tissue classes from a manually labelled training set. The technical details have been well described elsewhere^88^. The volumes of the striatal areas, caudate nucleus, putamen, and NAcc, and the eTIV were obtained from the statistical output. One subject in the NGC group was excluded from the FreeSurfer volumetric analysis due to the errors observed during processing.

### Statistical Analysis

Group comparisons of the demographic and clinical variables were conducted using a one-way analysis of variance (ANOVA) and two-sample t-test for demographic and clinical variables using IBM SPSS Statistics for Windows, version 20.0 (IBM SPSS, Armonk, NY, USA). Two-tailed *P* < 0.05 was considered to be statistically significant.

Whole brain voxel-wise comparisons of the GM density were performed using the analysis of covariance (ANCOVA) with age and IQ as nuisance covariates in SPM8 (*P*_FDR-corrected_ < 0.05). Then, subsequent post hoc t-tests were carried out to examine the inter-group difference with an uncorrected threshold of *P* < 0.001 with cluster extent threshold of *P*_FWE-corrected_ < 0.05 for multiple comparisons with a nonstationary smoothness correction^89^. The FreeSurfer volumetry analysis of the striatum was conducted using a multivariate ANCOVA with age and eTIV as covariates. Bonferroni correction was used for multiple comparisons (*P* < 0.0083; 0.05/6).

To explore the relationship between the structural measurements showing a group difference and the characteristics of the Internet gaming use, we merged the two groups of Internet gaming users (e.g., IGD and IGC groups) and performed a Pearson’s correlation analysis on the standardized variables. Additionally, we assessed whether the structural measurements (mediator variable) affected the relationship between lifetime usage of Internet gaming (causal variable) and depressed level (outcome variable) by performing a mediation analysis. These correlation and regression analyses were conducted in SPSS at the 5% significance level.
